# Atherosclerotic changes of the parent artery associated with ruptures in small (<7 mm) anterior communicating artery aneurysms

**DOI:** 10.3389/fneur.2026.1930043

**Published:** 2026-09-24

**Authors:** Nanhao Li, Xinjun Chen, Yutang Tan, Hao Chen, Yu Feng, Shen Yun Yuan, Wenyuan Zhao, Yihui Ma

**Affiliations:** Department of Neurosurgery, Zhongnan Hospital, Wuhan University, Wuhan, China

**Keywords:** AcoA, atherosclerosis, fluid–structure interaction, parent artery, small aneurysm

## Abstract

**Background:**

Small unruptured aneurysms are usually considered low-risk lesions and are typically managed conservatively. However, recent evidence indicates that small aneurysms account for a substantial proportion of ruptured intracranial aneurysms.

**Objective:**

To determine which morphology- and hemodynamics-related parameters are associated with the rupture status of small (<7 mm) anterior communicating artery (ACoA) aneurysms.

**Methods:**

We retrospectively reviewed 332 consecutive patients with ACoA aneurysms who were treated at our institute. Clinical data, imaging data, and intraoperative video recordings were reviewed and analyzed.

**Results:**

In total, 317 patients with small (<7 mm) ACoA aneurysms were included in the study, among whom 220 (69.4%) presented with ruptured aneurysms and 97 (30.6%) with unruptured aneurysms. The ruptured aneurysms showed a significantly higher height-to-width ratio, aspect ratio, and size ratio than the unruptured aneurysms (*p* < 0.05), and were more often irregular in shape or accompanied by a daughter sac. Among the 317 aneurysms, 53 aneurysms were analyzed for intracranial atherosclerosis and 47 aneurysms were evaluated to assess the hemodynamic parameters. Moderate-to-severe parent-artery atherosclerosis was also more significantly found in the ruptured aneurysms group (*p* < 0.01). Fluid–structure interaction analysis showed that, compared with the unruptured aneurysms, the ruptured aneurysms were associated with a lower time-averaged wall shear stress, higher oscillatory shear index, and higher von Mises stress in the parent artery.

**Conclusion:**

Our study demonstrated that the rupture of small (<7 mm) ACoA aneurysms may be associated with the parent artery and aneurysmal neck atherosclerosis, the hemodynamic environment, and adverse morphological characteristics.

## Introduction

Advances in noninvasive vascular imaging have led to a substantial increase in the detection of unruptured intracranial aneurysms (UIAs), particularly via computed tomography angiography (CTA) and magnetic resonance angiography. The incidence of UIAs has been reported to range from approximately 3.2 to 7% of adults (1, 2). While many such aneurysms remain asymptomatic, their rupture could lead to subarachnoid hemorrhage (SAH), which carries a high risk of death and permanent neurological impairment (3). In routine practice, most incidentally detected aneurysms tend to be small (4). Traditionally, small UIAs have been viewed as low-risk lesions, and conservative management with periodic imaging is commonly selected as the treatment modality. Nevertheless, there is growing clinical evidence that questions this size-based approach. In particular, several SAH cohorts have shown that a large proportion of ruptured aneurysms are below the conventional 7-mm threshold, with some studies reporting that approximately 75% of ruptured aneurysms are <7 mm (5, 6). For instance, Jagadeesan et al. found that ruptured posterior communicating artery aneurysms were predominantly small, with many even smaller than 3 mm (7). These findings suggest that a small size alone does not reliably indicate stability of the aneurysm, and that treatment decisions for small UIAs remain challenging and require careful consideration of a range of factors.

Beyond the aneurysm size, there is a growing body of evidence suggesting that the rupture risk is influenced by the lesion geometry and local flow conditions. Parameters such as the size ratio (SR), aspect-related indices, and wall shear stress (WSS) have been linked to the rupture status of intracranial aneurysms (8–10). Nevertheless, evidence specific to small anterior communicating artery (ACoA) aneurysms remains limited, despite the recognized rupture tendency of aneurysms in this location (11, 12). In the present study, we evaluated the imaging-derived morphological and hemodynamic features of small (<7 mm) ACoA aneurysms, paying particular attention to how parent-vessel atherosclerotic remodeling may interact with local biomechanical stress at the aneurysm site.

## Materials and methods

### Patient population

We retrospectively screened all patients who underwent treatment for ACoA aneurysms at our institute from January 2018 to October 2025, following study approval from the institutional review committee. Case identification was based on the institute’s electronic medical records, yielding 332 consecutive patients with 338 ACoA aneurysms. All the patients had undergone angiographic evaluation by CTA or digital subtraction angiography (DSA). In those with SAH, head CT was used to document the hemorrhage. The final study population consisted of 317 patients with a single small ACoA aneurysm, defined as a maximum aneurysm diameter of <7 mm.

### Definition of the morphological features

Three-dimensional CTA or DSA datasets were used to assess the aneurysm morphology and A1 segment anatomy. The evaluated morphological variables included the aneurysm size, neck diameter, aneurysm width, aneurysm height, parent-vessel diameter, size ratio (SR), aspect ratio (AR), and A1 segment configuration. The aneurysm size was measured as the greatest distance from the aneurysm neck plane to the dome. Aneurysm neck diameter was the largest cross-sectional diameter of the aneurysm neck. The width of the aneurysm was defined as the maximum distance of the line connecting the sides of the aneurysm wall. The aneurysm height was measured between the center of the aneurysm neck and the greatest distance to the aneurysm dome. In the anterior complex, it was defined as A1 hypoplasia (size of the A1 segment of the ACA 50% of the contralateral dominant A1 segment) or aplasia of A1 (absence of the A1 segment of the ACA). The aspect ratio (AR) was calculated by dividing the maximum perpendicular height of the aneurysm by the mean neck diameter. The parent-artery diameter was determined using two measurements: D1, obtained at the aneurysm neck perpendicular to the direction of flow, and D2, obtained perpendicular to the flow at a distance of 1.5 × D1 from the D1 site. The average parent-artery diameter was calculated as (D1 + D2)/2, and the size ratio (SR) was defined as the maximum aneurysm height divided by this average diameter. The height-to-width ratio (H/W) was calculated as the maximum perpendicular height divided by the aneurysm width. These measurements were obtained according to previously reported definitions, as outlined below (9, 13). All the morphological parameters were evaluated and confirmed by two experienced neurosurgeons (Yihui Ma and Xinjun Chen, both of whom have more than 10 years of working experience).

The flow angle (FA) was measured as the angle formed by the aneurysm height axis and the parent vessel. Aneurysm projection was classified as superior, anterior, or inferior. For this classification, the reference axis was defined as the line connecting the origin of the dominant A1 segment to the aneurysm base. The line from the aneurysm base to the dome was then used to determine the direction of projection: anterior if located in front of the reference axis, inferior if below it, and superior if above it. The dome morphology was recorded as either regular or irregular (Figure 1). An irregular morphology was defined according to the presence of a bilobulated or multilobulated bleb, daughter sac, or focal wall protrusion (8).

**Figure 1:**
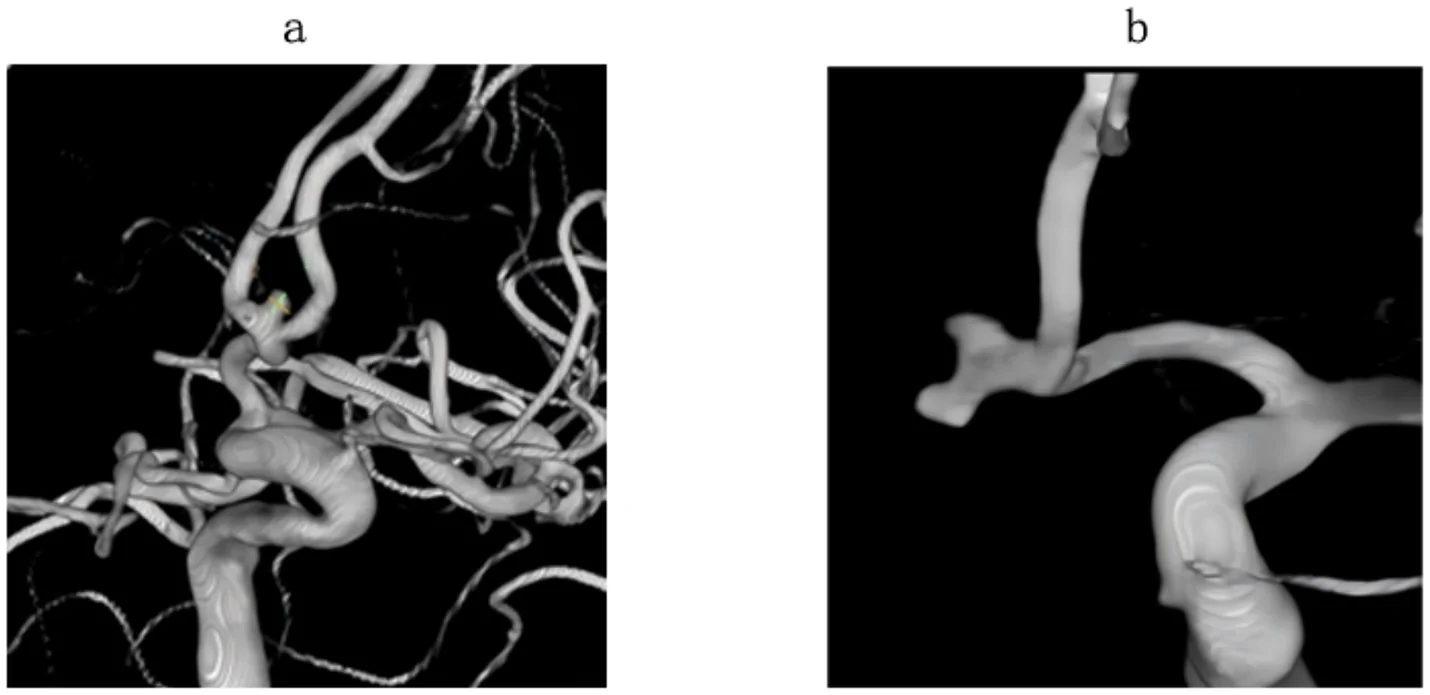
Example of anterior communicating artery aneurysm (ACoA) morphology determined by three-dimensional digital subtraction angiography. **(a)** An example of regular ACoA. **(b)** An example of irregular ACoA with daughter sac.

### Calculation of the hemodynamic parameters

In this study, the hemodynamic parameters of the parent artery and aneurysm wall were calculated by a fluid–structure interaction (FSI) simulation method (Figure 2). Hemodynamic assessment was performed for both the parent artery and aneurysm wall using fluid–structure interaction (FSI) modeling, as illustrated in Figure 2. Based on pulsatile-flow modeling, the analyzed parameters included the normalized time-averaged WSS (TAWSS), oscillatory shear index (OSI), von Mises stress (VMS), and relative residence time (RRT) (14).

**Figure 2:**
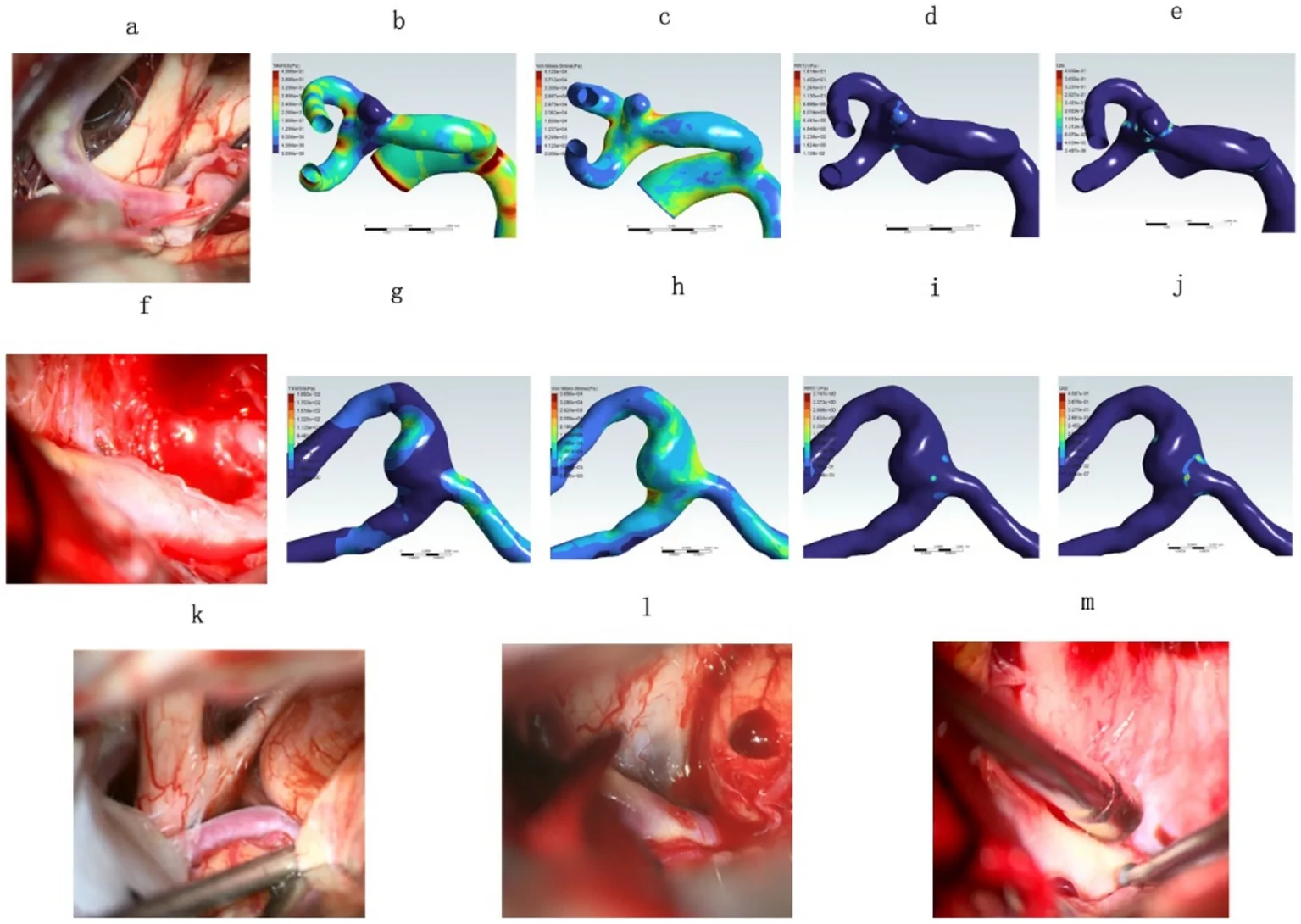
Example intraoperative photomicrographs and the results from fluid–structure interaction (FSI) analysis of the parent artery and aneurysm wall. **(a)** Intraoperative view of an unruptured intracranial aneurysm showing a mild atherosclerotic lesion on the parent artery. **(b–e)** FSI analysis of the parent artery and aneurysm wall of the unruptured aneurysm. **(f)** Intraoperative view of a ruptured intracranial aneurysm showing severe atherosclerotic lesions on the parent artery. **(g–j)** FSI analysis of the parent artery and aneurysm wall of the ruptured aneurysm. **(k–m)** Intraoperative photomicrographs showing the severity of arteriosclerosis in the parent artery (normal, mild, or severe).

CTA and DSA raw data were exported in DICOM format and imported into Mimics 21.0 (Materialise, Belgium). Vascular masks were generated by threshold segmentation and refined to retain the target regions (ACoA aneurysm, A1/A2 segments of the anterior cerebral arteries, communicating segment of the internal carotid artery, and proximal M1 segment of the middle cerebral artery). The masks were smoothed in 3-matic (Materialise, Belgium), and a 0.3 mm vessel wall was constructed. The inlet and outlet sections were trimmed, and the model was exported as STL to SpaceClaim (ANSYS, USA), where separate fluid and solid domains were created and connected via share topology. Meshing was performed in ANSYS Meshing. Eight prism layers (growth rate 1.2) were generated near the wall, with a global element size of 0.15 mm. The surface was discretized with triangle meshes and the volume with tetrahedral elements, with skewness < 0.85 and orthogonal quality > 0.15. Fluid domain: Blood was modeled as an incompressible non-Newtonian fluid (density 1,060 kg/m^3^) using the Carreau viscosity model (η₀ = 0.056 Pa·s,η∞ = 0.00345 Pa·s, λ = 3.313 s, *n* = 0.3568). Solid domain: The vessel wall was modeled as an isotropic linear elastic material (Young’s modulus 1.7 MPa, Poisson’s ratio 0.45, density 1,100 kg/m^3^). Solver settings: Simulations were performed using ANSYS Fluent 2025 R1 with two-way fluid–structure interaction and dynamic mesh method (diffusion-based smoothing + local remeshing). A transient solver was used with a time step of 0.01 s, maximum 40 coupling iterations per step, and 300 total steps (3 cardiac cycles). Inlet velocity and outlet pressure boundaries were adopted from previous study (15). Post-processing: Parameters were extracted in CFD-Post. TAWSS was obtained by time-averaging wall shear stress. OSI and RRT were calculated directly via expressions. Von Mises stress was extracted as the time-averaged equivalent stress over one cardiac cycle. All parameters were extracted separately for the parent artery and the aneurysm sac.

### Intraoperative qualitative assessment of the aneurysm wall features

Cases in which the aneurysm wall and parent artery could be observed sufficiently in intraoperative images were included in the present study and analyzed. Microsurgical clipping was performed in all cases using a Pentero 900 surgical microscope (Carl Zeiss Microscopy GmbH, Jena, Germany). The approach most often used for ACoA aneurysms is the pterional approach. The available intraoperative inspection is performed circumferentially for anterior cerebral artery aneurysms and semicircumferentially for ACoA aneurysms. Neurosurgeons then evaluate the parent artery and aneurysm wall features. The degree of atherosclerotic changes was reviewed and assessed by two skilled neurosurgeons together (Yihui Ma and Xinjun Chen, both of them have working experience more than 10 years). Red-walled aneurysm refers to intracranial aneurysms that appear visibly red, or thin-walled without arteriosclerosis during microsurgical dissection. The severity of arteriosclerosis in the parent artery is classified into three grades (Normal: no atherosclerotic plaque; mild: less than half of the parent artery segment is atherosclerotic; severe: more than half of the parent artery segment is atherosclerotic). A white or yellow aneurysm wall is considered a sign of arteriosclerosis of the aneurysm wall (Figure 2).

### Statistical analysis

All analyses were performed using SPSS 26.0 (IBM SPSS, Armonk, NY, USA). Normality was assessed using the Shapiro–Wilk test. Normally distributed continuous variables were expressed as mean ± SD and compared using the independent-sample t-test; non-normally distributed variables were expressed as median (IQR) and compared using the Mann–Whitney U test. Categorical variables were expressed as counts and compared using the χ^2^ test, or Fisher’s exact test when the expected frequency in any cell was <5. Due to the zero-cell issue, Fisher’s exact test was used, and Haldane’s continuity correction (+0.5 to each cell) was applied to estimate OR and 95% CI. As most FSI parameters deviated from normal distribution,all between-group comparisons were performed using the Mann–Whitney U test, with data presented as median (IQR). All tests were two-sided, and *p* < 0.05 was considered statistically significant.

## Results

### Patients’ baseline characteristics

A total of 317 patients with small ACoA aneurysms (<7 mm) were included, comprising 220 ruptured and 97 unruptured cases. As shown in Table 1, patients in the ruptured group were significantly younger than those in the unruptured group (56.52 ± 9.93 vs. 59.97 ± 8.61 years, *p* = 0.003). Alcohol consumption (37.27% vs. 19.58%, *p* < 0.001) was significantly more prevalent in the ruptured group. Aneurysm size was larger in the ruptured group (4.14 ± 1.02 mm vs. 3.75 ± 1.41 mm, *p* = 0.015). Regarding morphological parameters, the ruptured group exhibited significantly higher AR (1.59 ± 0.46 vs. 1.22 ± 0.47, *p* < 0.001), H/W (1.49 ± 0.43 vs. 1.02 ± 0.31, *p* < 0.001), and SR (2.11 ± 0.65 vs. 1.66 ± 0.85, *p* < 0.001). Daughter sac formation (48.18% vs. 16.49%, *p* < 0.001) and irregular aneurysm (75.45% vs. 31.96%, *p* < 0.001) were more frequent in the ruptured group. No significant differences were observed in sex, hypertension, diabetes, aneurysm-parent artery angle, ipsilateral A1-A2 angle, A1 segment configuration, or aneurysm projection (*p* > 0.05). Although smoking (30.91% vs. 22.68%, *p* = 0.134) and superior projection (63.64% vs. 49.48%, *p* = 0.052) was more common among the ruptured aneurysms, the difference between the groups was borderline and did not reach statistical significance.

**Table 1:** Clinical, morphological, and vascular features of ruptured vs. unruptured anterior communicating artery aneurysms.

Variable	Ruptured (*n* = 220)	Unruptured (*n* = 97)	OR(95%Cl)	*p*-value
Age, y	56.52 ± 9.93	59.97 ± 8.61		0.003
Male Sex, n (%)	112 (50.91%)	46 (47.42%)	1.150 (0.713 to 1.855)	0.567
Hypertension, n (%)	132 (60.00%)	63 (64.95%)	0.810 (0.493 to 1.330)	0.404
Diabetes, n (%)	20(9.09%)	10 (10.31%)	0.870 (0.391 to 1.936)	0.733
Smoking, n (%)	68 (30.91%)	22 (22.68%)	1.525 (0.876 to 2.656)	0.134
Alcohol, n (%)	82(37.27%)	19(19.58%)	2.439 (1.378 to 4.318)	0.002
Aneurysm size, mm	4.14 ± 1.02	3.75 ± 1.41		0.015
Aneurysm height, mm	4.10 ± 1.04	3.33 ± 1.40		<0.001
Aneurysm width, mm	2.90 ± 0.93	3.35 ± 1.37		0.004
Neck size, mm	2.70 ± 0.79	2.80 ± 1.01		0.384
AR	1.59 ± 0.46	1.22 ± 0.47		<0.001
H/W	1.49 ± 0.43	1.02 ± 0.31		<0.001
SR	2.11 ± 0.65	1.66 ± 0.85		<0.001
Aneurysm with daughter sac, n (%)	106 (48.18%)	16 (16.49%)	4.707 (2.589 to 8.559)	<0.001
Irregular aneurysm shape, n (%)	166 (75.45%)	31(31.96%)	6.530 (3.876 to 11.111)	<0.001
Aneurysm-parent artery angle	128.18 ± 25.19	127.53 ± 26.88		0.836
Ipsilateral A1-A2 angle	98.37 ± 22.97	101.25 ± 22.64		0.302
A1 segment configuration				0.31
Well-developed (Bilateral)	49 (22.27%)	26 (26.80%)		
Unilateral hypoplasia	90 (40.91%)	31 (31.96%)		
Unilateral aplasia	81 (36.82%)	40 (41.24%)		
Bilateral A1 ratio (Non-dominant/Dominant)				0.495
> 50%	85 (38.64%)	38 (39.18%)		
< 50%	58 (26.36%)	20 (20.62%)		
No A1	77 (35.00%)	39 (40.21%)		
Aneurysm projection				0.052
Superior	140 (63.64%)	48 (49.48%)		
Inferior	56 (25.45%)	32 (32.99%)		
Anterior	24 (10.91%)	17 (7.53%)		

AR: Aspect ratio; H/W: Height-width ratio; SR: Size ratio.

### Morphological characteristics of the aneurysms

To evaluate collinearity among morphological continuous variables, Spearman’s rank correlation analysis was performed for aneurysm size, AR, H/W, and SR in the full cohort (Table 2). SR was strongly correlated with aneurysm size (rₛ = 0.755, *p* < 0.001), indicating substantial collinearity. AR showed moderate correlations with SR (rₛ = 0.467, *p* < 0.001) and aneurysm size (rₛ = 0.336, *p* < 0.001). H/W demonstrated relatively weaker correlations with all indices (rₛ = 0.187–0.568). Accordingly, only AR was retained as the representative morphological variable in the subsequent multivariable regression analyses.

**Table 2:** Spearman correlation matrix among morphological indices.

Variable	AR	H/W	SR	Aneurysm size
AR	1	0.568***	0.467***	0.336***
H/W	—	1	0.384***	0.187***
SR	—	—	1	0.755***
Aneurysm Size	—	—	—	1

AR: Aspect ratio; H/W: Height-width ratio; SR: Size ratio. ***p < 0.001.

To identify independent risk factors for aneurysm rupture, variables with *p* < 0.05 in univariate analysis and clinically recognized confounders (age, alcohol, AR, daughter sac, and irregular morphology) were entered into a multivariable logistic regression model using the Enter method (Table 3). AR (OR = 6.584, 95% CI: 2.988–14.505, *p* < 0.001), alcohol (OR = 2.110, 95% CI: 1.098–4.065, *p* < 0.05), and irregular morphology (OR = 6.084, 95% CI: 2.680–13.808, *p* < 0.001) were identified as independent risk factors for rupture. Patients in the rupture group exhibited a significant younger age distribution (OR = 0.945, 95% CI: 0.915–0.977, *p* < 0.001). Daughter sac did not reach statistical significance after adjustment (*p* > 0.05).

**Table 3:** Multivariable logistic regression analysis for rupture of small ACoA aneurysms.

Variables	Univariate analysis		Multivariate analysis	
	OR (95% CI)	*p* value	OR (95% CI)	P value
Age		0.003	0.945 (0.915–0.977)	< 0.001
Alcohol	2.439 (1.378–4.318)	0.002	2.110 (1.098–4.065)	0.025
Aspect ratio (AR)		< 0.001	6.584 (2.988–14.505)	< 0.001
Daughter sac	4.707 (2.589–8.559)	< 0.001	1.364 (0.601–2.245)	0.458
Irregular morphology	6.530 (3.876–11.111)	< 0.001	6.084 (2.680–13.808)	< 0.001

AR: Aspect ratio.

### Atherosclerotic and hemodynamic characteristics

Among the 317 aneurysms analyzed in the study, 32 ruptured and 21 unruptured aneurysms were evaluated for intracranial atherosclerosis (Table 4). Age, hypertension, diabetes, smoking, alcohol consumption and sex distribution were comparable between the ruptured and unruptured groups. Parent-artery atherosclerosis was observed in 9 patients in the ruptured group (28.13%) but in none of the unruptured patients, indicating its significantly higher prevalence in ruptured aneurysms (*p* < 0.01). In contrast, ICA atherosclerosis was similarly distributed between the two groups, occurring in 17 ruptured cases (53.13%) and 11 unruptured cases (52.38%). Atherosclerotic changes at the aneurysm neck were also more frequent in the ruptured small (<7 mm) ACoA aneurysms. By comparison, aneurysm wall atherosclerosis was identified in 11 ruptured cases (34.38%) and 12 unruptured cases (57.14%), with no statistically significant between-group difference. Rupture status was significantly associated with aneurysm-neck atherosclerosis in the small (<7 mm) ACoA aneurysms. However, the prevalence of aneurysm wall atherosclerosis did not differ significantly between the ruptured and unruptured aneurysms, despite its numerically lower prevalence in the ruptured group (34.38% vs. 57.14%).

**Table 4:** Comparison of atherosclerosis-related risk factors between ruptured and unruptured intracranial aneurysm groups.

Variable	Ruptured (*n* = 32)	Unruptured (*n* = 21)	OR(95%Cl)	*p*-value
Age, y	57.56 ± 8.14	59.86 ± 6.06		0.274
Male sex, n (%)	18 (56.25%)	11 (52.38%)	0.856(0.283 to 2.583)	0.782
Hypertension, n (%)	23 (71.88%)	14 (66.67%)	1.278(0.389 to 4.202)	0.686
Diabetes, n (%)	3 (9.38%)	3 (14.29%)	0.621(0.113 to 3.414)	0.581
Smoking, n (%)	9 (28.13%)	3 (14.29%)	2.348(0.554 to 9.958)	0.239
Alcohol, n (%)	14 (43.75%)	4 (19.05%)	3.306(0.906 to 12.055)	0.063
Aneurysm size	4.18 ± 1.25	3.78 ± 1.40		0.278
Atherosclerosis of parent artery, n (%)	9 (28.13%)	0 (0.00%)	17.38(0.95 to 316.6)	0.008
Aneurysm neck sclerosis, n (%)	24 (75%)	9 (42.86%)	4.0(1.232 to 12.989)	0.018
Aneurysm wall atherosclerosis, n (%)	11 (34.38%)	12 (57.14%)	0.393(0.127 to 1.217)	0.102
ICA atherosclerosis, n (%)	17 (53.13%)	11 (52.38%)	1.030(0.342 to 3.103)	0.958

ICA, Internal carotid artery.

Among the 317 aneurysms analyzed in the study, 25 ruptured and 22 unruptured aneurysms were evaluated to assess the hemodynamic parameters (Table 5). In the parent artery, the ruptured group showed significantly lower TAWSS (min) (0.39 Pa [0.28–0.56] vs. 1.08 Pa [0.51–1.95], *p* < 0.001), significantly higher OSI (max) (0.34 [0.33–0.41] vs. 0.09 [0.02–0.27], *p* < 0.001), significantly higher RRT (max) (10.82 1/Pa [4.69–22.97] vs. 2.39 [0.74–5.96], *p* < 0.001), and significantly higher mean Von Mises stress (41,392 Pa [30,400.5–54,037] vs. 24,600 Pa [18,515–27,456.5], *p* < 0.001). In the aneurysm sac, only mean Von Mises stress was significantly higher in the ruptured group (26,654 Pa [19,464.5–31,098] vs. 19,012.5 Pa [16,220.75–23,544.75], *p* = 0.021). No significant differences were observed for TAWSS (max) in either location, OSI (max), or RRT (max) in the aneurysm sac (*p* > 0.05). These findings are exploratory and require validation in larger cohorts.

**Table 5:** Comparison of baseline characteristics and hemodynamic parameters between ruptured and unruptured groups.

Variable	Ruptured (*n* = 25)	Unruptured (*n* = 22)	*P*-value
Age, y	57.00 ± 7.42	60.68 ± 6.96	0.087
Male sex, n (%)	13 (52%)	11 (50%)	0.891
Hypertension, n (%)	20 (80%)	13 (59.09%)	0.118
Diabetes, n (%)	2 (8%)	2 (9.09%)	0.894
Smoking, n (%)	7 (28%)	6 (27.27%)	0.956
Alcohol, n (%)	10 (40%)	4 (18.18%)	0.103
Aneurysm size	4.15 ± 1.21	3.90 ± 1.32	0.487
Parent artery TAWSS (max)	14.37 [10.24, 22.60]	15.10 [9.53, 18.44]	0.601
Parent artery TAWSS (min)	0.39 [0.28, 0.56]	1.08 [0.51, 1.95]	<0.001
Parent artery OSI (max)	0.34 [0.33, 0.41]	0.09 [0.02, 0.27]	<0.001
Parent artery RRT (max)	10.82 [4.69, 22.97]	2.39 [0.74, 5.96]	<0.001
Parent artery Mean Von mises stress (max)	41,392 [30400.5, 54,037]	24,600 [18,515, 27456.5]	<0.001
Aneurysm TAWSS (max)	5.02 [2.56, 10.36]	7.84 [4.22, 10.48]	0.536
Aneurysm TAWSS (min)	0.35 [0.18, 0.88]	0.79 [0.23, 1.89]	0.172
Aneurysm OSI (max)	0.06 [0.03, 0.24]	0.24 [0.05, 0.32]	0.153
Aneurysm RRT (max)	4.56 [1.04, 7.93]	2.98 [1.40, 12.09]	0.773
Aneurysm Mean Von mises stress (max)	26,654 [19464.5, 31,098]	19012.5 [16220.75, 23544.75]	0.021

TAWSS, time-averaged wall shear stress; OSI, oscillatory shear index; RRT, relative residence time.

## Discussion

This study’s findings suggest there is a potential relationship between local atherosclerotic remodeling and ruptures in small (<7 mm) ACoA aneurysms, particularly when such changes involve the parent artery or aneurysm neck. Compared with red-walled aneurysms and the normal parent artery, both the parent artery (A1 segment) and the aneurysm neck with atherosclerotic changes had lower TAWSS, higher OSI, and higher VMS values in the present study. Morphological instability is another important feature of ruptured small ACoA aneurysms, as reflected by the higher frequency of daughter sacs and irregular shapes, together with increased AR, SR, and H/W ratios, in the rupture cases in this study.

Traditionally, the risk of rupture in intracranial aneurysms has been associated with larger aneurysms, with the size threshold of greater than 7 mm commonly cited as indicating a significant rupture risk (16, 17). This has led to widespread clinical practices involving more aggressive surveillance and intervention strategies for larger aneurysms (18). However, the findings of the present study challenge the traditional view that a small aneurysm size necessarily implies a low rupture risk. Indeed, prior SAH cohorts have demonstrated that many ruptured aneurysms are small (5, 9, 19). For instance, in the series reported by Forget et al., 94.4% of ruptured ACoA aneurysms were <10 mm, and 43.6% were <5 mm (20). Our data showed a similar pattern, wherein nearly all the ACoA aneurysms in our consecutive cohort were <7 mm in size, and two-thirds of the ruptured ACoA aneurysms also fell below this threshold. Therefore, this suggests that considering size alone may be insufficient for accurate risk stratification in ACoA aneurysms, particularly for lesions classified as small.

In our study, patients in the ruptured group were significantly younger than those in the unruptured group (adjusted OR = 0.946, 95% CI: 0.915–0.977, *p* < 0.001). The fascinating and counterintuitive finding likely does not mean that youth cause rupture, but rather that rupture is the primary mode of presentation in young patients due to higher hemodynamic forces, acute trigger exposure, and the absence of routine screening. Concurrently, older patients have a large reservoir of incidentally discovered, stable unruptured aneurysms that dilute the rupture proportion in that age group. In older populations, unruptured aneurysms are far more commonly discovered incidentally during routine brain imaging (MRI/CT) for other issues like stroke workups. Conversely, younger patients rarely undergo brain imaging unless they have acute, severe symptoms.

### Role of atherosclerotic changes

In the present study, the ruptured small (<7 mm) ACoA aneurysms showed more frequent atherosclerotic changes at the parent A1 segment and aneurysm neck. This local atherosclerotic remodeling appeared to coincide with disturbed parent-artery hemodynamics, as reflected by the lower TAWSS and higher OSI and VMS values. This suggests that parent vessel atherosclerosis is not merely a coexisting condition but may actively contribute to the pathogenesis of ruptures in small aneurysms. Specifically, when atherosclerotic changes occur in the parent vessel or at the aneurysm neck, they may disrupt the local flow patterns and create an unfavorable hemodynamic milieu, which can eventually lead to a rupture of the aneurysm. Mahdi et al. investigated the impact of atherosclerosis on hemodynamics, initiation, and growth of an IA using FSI analysis. They found that blood velocity and pressure throughout the vessels increase the risk of arterial tissue remodeling and aneurysm formation (21). Another study has also suggested that atherosclerosis may compromise the arterial wall integrity, thereby increasing the susceptibility to aneurysm development and rupture (22). In the present study, parent-artery atherosclerosis was more frequently observed in ruptured small aneurysms, especially in cases with more advanced atherosclerotic involvement. Furthermore, the specific association of ruptured aneurysms with atherosclerotic changes at the aneurysm neck points toward a localized vessel wall pathology. This finding supports the hypothesis that atherosclerotic degeneration at the neck may act as a nidus for wall instability, weakening the structural integrity at the critical interface between the parent vessel and the aneurysm sac. Notably, atherosclerotic changes of the aneurysm wall appeared to be numerically less common in the ruptured aneurysms than in the unruptured aneurysms, but this trend did not reach statistical significance. This may suggest that aneurysm wall atherosclerosis is a later-stage phenomenon in the degenerative process, whereas the hemodynamic consequences of the parent vessel disease play a more direct role in precipitating a rupture.

It has been shown that FSI simulation can provide a crucial link between observed morphological changes and the biomechanical forces acting on the intracranial artery and aneurysm wall (21). Using FSI, we found that the ruptured aneurysms were characterized by a significantly lower TAWSS (min) in the parent vessel, higher OSI, RRT, and higher VMS compared to red-walled aneurysms and parent arteries. This suggests that the magnitude of the shear stress, rather than its direction or maximum peak value, is the dominant hemodynamic feature distinguishing ruptured from unruptured small ACoA aneurysms. This observation is in line with previous evidence indicating that a low TAWSS may promote inflammatory responses, endothelial dysfunction, and matrix degradation through a process of endothelial cell mechanotransmission (23, 24).

After atherosclerosis of the parent artery, vascular compliance decreases, leading to frequent reversals or a high OSI. An increased OSI indicates more pronounced oscillatory shear, a condition that may favor endothelial injury, inflammation, and progressive remodeling of the parent artery and aneurysm neck. In parallel, atherosclerotic stiffening of the parent vessel and aneurysm neck may amplify wall stress under similar hemodynamic loading, which is consistent with the elevated VMS observed in the ruptured aneurysms in the present study. High wall stress can not only damage the aneurysm-bearing artery itself but may also be mechanically transmitted to the aneurysm neck and aneurysm top, becoming a key mechanical factor inducing rupture.

### Aneurysm size and morphological features

Our results confirm the well-established role of the aneurysm size in the risk of its rupture (25). In the present study, the ruptured and unruptured small (<7 mm) ACoA aneurysms differed significantly in terms of their AR, SR, and H/W ratio. In particular, a higher AR was found to be associated with the rupture status of small ACoA aneurysms. In this regard, a larger AR is thought to reflect an elongated and less stable shape of the aneurysm, which could predispose the aneurysm to rupture under increased hemodynamic stress (26). This result is consistent with previous studies demonstrating that a higher AR correlates with an increased rupture risk in intracranial aneurysms (27). Similarly, a higher SR was found to be associated with the rupture status in this cohort. A larger SR suggests a relatively larger aneurysm compared to the parent vessel, which would lead to increased mechanical stress on the aneurysm wall. This is consistent with previous research that showed that a larger SR may cause aneurysm rupture due to higher wall stress and the reduced stability of the aneurysm (12, 27).

Our results demonstrated that small (<7 mm) ACoA aneurysms are not a homogeneous group; their morphology is critical. Ruptured small ACoA aneurysms also tend to have more complex morphologies, including irregular contours and daughter sacs. In addition, a superior projection was observed more often in the ruptured lesions in our cohort, suggesting a possible projection-related hemodynamic influence, although the between-group difference was only borderline. These features are an indication of focal wall weakness and unstable hemodynamics. In particular, an irregular shape or a daughter sac represents a focal point of wall stress and is often a sign of impending instability (28, 29). Furthermore, the direction of projection likely influences the impact of inflow jets from the parent A1 arteries, creating areas of high wall shear stress, which can trigger inflammatory degradation of the wall. A superior projection may influence the local inflow patterns and hemodynamic loading at the aneurysm wall. Although this finding did not reach statistical significance in the present study, the coexistence of an irregular morphology and daughter sac formation may indicate a more unstable lesion phenotype. Therefore, small ACoA aneurysms with these features may warrant closer clinical surveillance and individualized treatment consideration, even when their maximum diameter is below 7 mm.

Conversely, the lack of significant differences in the aneurysm neck size, aneurysm-parent artery angle, and A1 segment configuration between the two groups in this study suggests that while these factors may influence aneurysm initiation or growth, they may not be the primary drivers of ruptures in ACoA aneurysms. Our findings suggest that once an aneurysm has formed, the local hemodynamics determined by the sac’s shape (considering also the AR and SR) may be more critical for its stability.

### Study limitations

This study has several limitations that should be considered when interpreting the findings. Most importantly, its retrospective design makes it vulnerable to selection bias and residual confounding. Second, while morphological factors are important, they do not fully account for the complex nature of aneurysm ruptures, which may also be influenced by genetic, biochemical, and environmental factors. Finally, several limitations inherent to our computational modeling should be acknowledged. First, we employed an isotropic linear elastic material model for the vessel wall, a commonly adopted simplifying assumption in intracranial aneurysm FSI studies. However, the actual arterial wall is a three-layered structure (intima, media, and adventitia) with anisotropic, nonlinear, and viscoelastic properties due to the orientation of collagen and elastin fibers. Second, we assumed a uniform wall thickness of 0.3 mm for all patients and all arterial segments. In reality, aneurysm walls exhibit non-uniform thickness distributions. Third, our inlet velocity and outlet pressure waveforms were adopted from published experimental data rather than patient-specific measurements. While literature-based boundary conditions are widely used in similar FSI studies, patient-specific flow conditions may yield more accurate hemodynamic predictions.

## Conclusion

Our study demonstrated that the rupture of small (<7 mm) ACoA aneurysms may be associated with the parent artery and aneurysmal neck atherosclerosis, and a hemodynamic environment characterized by a lower TAWSS, higher OSI, and higher VMS. The rupture of small (<7 mm) ACoA aneurysms was found to be associated with a combination of a larger size and, more importantly, with certain unfavorable morphological features, including an irregular configuration, daughter sac formation, and increased AR, SR, and H/W values. These results suggest that rupture assessment in small ACoA aneurysms should not rely on the aneurysm size alone, but should also incorporate a consideration of the vessel-wall changes, aneurysm morphology, and hemodynamic characteristics to support more individualized clinical decision-making.

## Data Availability

The raw data supporting the conclusions of this article will be made available by the authors, without undue reservation.
